# An Informatics Framework to Assess Consumer Health Language Complexity Differences: Proof-of-Concept Study

**DOI:** 10.2196/16795

**Published:** 2020-05-21

**Authors:** Biyang Yu, Zhe He, Aiwen Xing, Mia Liza A Lustria

**Affiliations:** 1 Florida State University School of Information Tallahassee, FL United States; 2 Florida State University Department of Statistics Tallahassee, FL United States

**Keywords:** consumer health informatics, readability, digital divide, health literacy

## Abstract

**Background:**

The language gap between health consumers and health professionals has been long recognized as the main hindrance to effective health information comprehension. Although providing health information access in consumer health language (CHL) is widely accepted as the solution to the problem, health consumers are found to have varying health language preferences and proficiencies. To simplify health documents for heterogeneous consumer groups, it is important to quantify how CHLs are different in terms of complexity among various consumer groups.

**Objective:**

This study aimed to propose an informatics framework (consumer health language complexity [CHELC]) to assess the complexity differences of CHL using syntax-level, text-level, term-level, and semantic-level complexity metrics. Specifically, we identified 8 language complexity metrics validated in previous literature and combined them into a 4-faceted framework. Through a rank-based algorithm, we developed unifying scores (CHELC scores [CHELCS]) to quantify syntax-level, text-level, term-level, semantic-level, and overall CHL complexity. We applied CHELCS to compare posts of each individual on online health forums designed for (1) the general public, (2) deaf and hearing-impaired people, and (3) people with autism spectrum disorder (ASD).

**Methods:**

We examined posts with more than 4 sentences of each user from 3 health forums to understand CHL complexity differences among these groups: 12,560 posts from 3756 users in Yahoo! Answers, 25,545 posts from 1623 users in AllDeaf, and 26,484 posts from 2751 users in Wrong Planet. We calculated CHELCS for each user and compared the scores of 3 user groups (ie, deaf and hearing-impaired people, people with ASD, and the public) through 2-sample Kolmogorov-Smirnov tests and analysis of covariance tests.

**Results:**

The results suggest that users in the public forum used more complex CHL, particularly more diverse semantics and more complex health terms compared with users in the ASD and deaf and hearing-impaired user forums. However, between the latter 2 groups, people with ASD used more complex words, and deaf and hearing-impaired users used more complex syntax.

**Conclusions:**

Our results show that the users in 3 online forums had significantly different CHL complexities in different facets. The proposed framework and detailed measurements help to quantify these CHL complexity differences comprehensively. The results emphasize the importance of tailoring health-related content for different consumer groups with varying CHL complexities.

## Introduction

### Background

The language gap between laypersons (health consumers) and health care professionals has been long recognized as the main hindrance to effective health communication and health information comprehension [1-3]. When interpreting health documents written mainly in professional language, consumers often depend on their own language to *fill in* the comprehension gap (eg, *depression* vs *depressive disorder*), which might lead to misinterpretation. Accordingly, it has also been widely agreed that health consumers should be given access to resources in their own languages [3-6]. To improve the readability of health-related content for average health consumers, there has been increasing interest in examining consumer health vocabularies [2,7], health readability measurement [8-10], and automated health text simplification approaches [11-14]. Studies on consumer health vocabularies have largely focused on extracting and building a terminology system of lay health terms used by average health consumers [2,7]. Health readability assessments have focused on developing linguistic metrics to quantify the text complexity of health content generated by health experts and professionals [9,13,15,16]. On the basis of the findings in both areas, automated health text simplification usually focuses on simplifying difficult texts with respect to 1 or 2 aspects (eg, medical jargon, long sentences) [1,11,12,14,18,19].

However, without a comprehensive understanding of the complexity difference between professional health language and consumer health language (CHL), current automated simplification approaches are inadequate to accurately determine what needs to be simplified and to what extent they should be simplified. Also, current simplification approaches assume that consumers share the same CHL preferences and that simplifying text to its lowest complexity can satisfy all users. For example, in synonym replacement tasks, researchers typically identify difficult medical words and then replace them with easier synonyms [12,19]. These one-size-fits-all automated simplification approaches ignore the diverse simplification needs of different health customers. Research suggests that consumers with varying health literacy levels have different CHL preferences [20-22]. In addition, contextual and sociocultural factors are found to affect the language preferences of different consumer groups to think, express, and communicate health-related topics [3]. For example, compared with average health consumers, cancer patients would be more familiar with cancer-related professional health terms (eg, genetic predisposition). Another drawback of this one-size-fits-all approach is that simplifying health content by replacing terms with lay alternatives with the lowest complexity may affect information accuracy and may inadvertently increase the length of the text [23]. In other words, an adaptive simplification approach that can balance simplicity, accuracy, and sentence length for user groups with various CHL preferences is ideal.

In this paper, CHL has been defined as a system of vocabularies, expressions, and grammar that is commonly used by a group of health consumers in thinking, expressing, and communicating their health-related topics. CHL complexity is defined as a combined measure of varying linguistic metrics, each of which quantifies the complexity of one linguistic feature of a CHL (eg, semantics, syntax, term). The goal of adaptive health text simplification is to simplify the professional health language used in Web-based health content to match the CHL complexities of targeted consumer groups. To quantify the CHL complexity differences for simplification purposes, the linguistic complexities of CHLs used by various health consumer groups should be investigated. The increasing availability of user-generated Web-based health communications (eg, blogs, online communities, social question and answer [Q&A] websites), provides us with ample opportunities to assess CHL complexity through automated text analysis [2,7,24].

Studies focused on health readability assessment typically quantify the complexity of Web-based health content written by health professionals for health consumers [25-27]. Researchers have developed complexity metrics that utilize a combination of various extracted linguistic features to assess the complexity of Web-based health content [9,13,16]. The metrics utilized in previous literature can be categorized into 4 groups, namely, text-level complexity (eg, syllables per word) [16,28], syntax-level complexity (eg, distributions of parts of speech [POS]) [16,29], term-level complexity (eg, density of professional medical terms) [15,16], and semantic-level complexity (eg, diversity of semantics) [15]. Examining how these linguistic features differ among various CHLs can help us gain a more accurate and comprehensive understanding of CHL complexity.

### Objectives

In this proof-of-concept study, we developed an informatics framework (consumer health language complexity [CHELC]) to assess CHL complexity based on existing health text readability metrics and apply this framework to explore complexity differences in CHL in 3 online forums designed for the general public, deaf and hearing-impaired people, and people with autism spectrum disorder (ASD). In previous studies, the latter 2 groups have been found to have relatively low health literacy [30-33], different language use behaviors [34,35], and limited access to adaptive health information services [36]. People with ASD were found to be repetitive and expressive by composing long sentences and words on the Web [35,37,38]. Pollard and Barnett [39] found that even highly educated deaf adults showed significant difficulty in understanding health vocabularies used in the Rapid Estimate of Adult Literacy in Medicine test. In addition, compared with the general population, deaf and hearing-impaired people exhibit significantly lower levels of health literacy and health knowledge [32]. Accordingly, ASD and deaf and hearing-impaired user groups might use less complex CHL, especially less complex health terms in their expressions. Motivated by these observations, in this study, we explore the use of different measures to assess CHL complexity and provide insights for the development of adaptive health text simplification tools to address the needs of various consumer groups.

We formulated 2 research questions (RQs) in this study:

- RQ1: What is the feasibility of using CHELC, which combines text-level, syntax-level, term-level, and semantic-level measures for examining CHL complexity among users in 3 distinct online forums designed for the general public, people with ASD, and deaf and hearing-impaired people?
- RQ2: How do the CHLs of users in online forums designed for the general public, people with ASD, and deaf and hearing-impaired people differ in complexity on the text level, syntax level, term level, and semantic level?

## Methods

### Consumer Health Language Complexity Measurement Framework

We built CHELC to incorporate a comprehensive array of linguistic complexity metrics developed in previous research. In this framework, we incorporated metrics of text-level, syntax-level, term-level, and semantic-level CHELC scores (CHELCS) to compare various CHLs through a rank-based algorithm. The overall complexity of CHL (CHELCS_overall_) was defined as the average value of 4 complexity scores.

We systematically reviewed the metrics that have been utilized in health readability and complexity assessment studies and comprehensively included credible metrics from all facets of linguistic measures. We performed the search on PubMed using the search terms of *health readability* to retrieve relevant articles and abstracts, which returned 3605 full-text articles to be screened. After excluding duplicates, non-English articles, and articles not about health readability evaluation or assessment, 9 studies with different assessment metrics were identified (Table 1).

**Figure 1:**
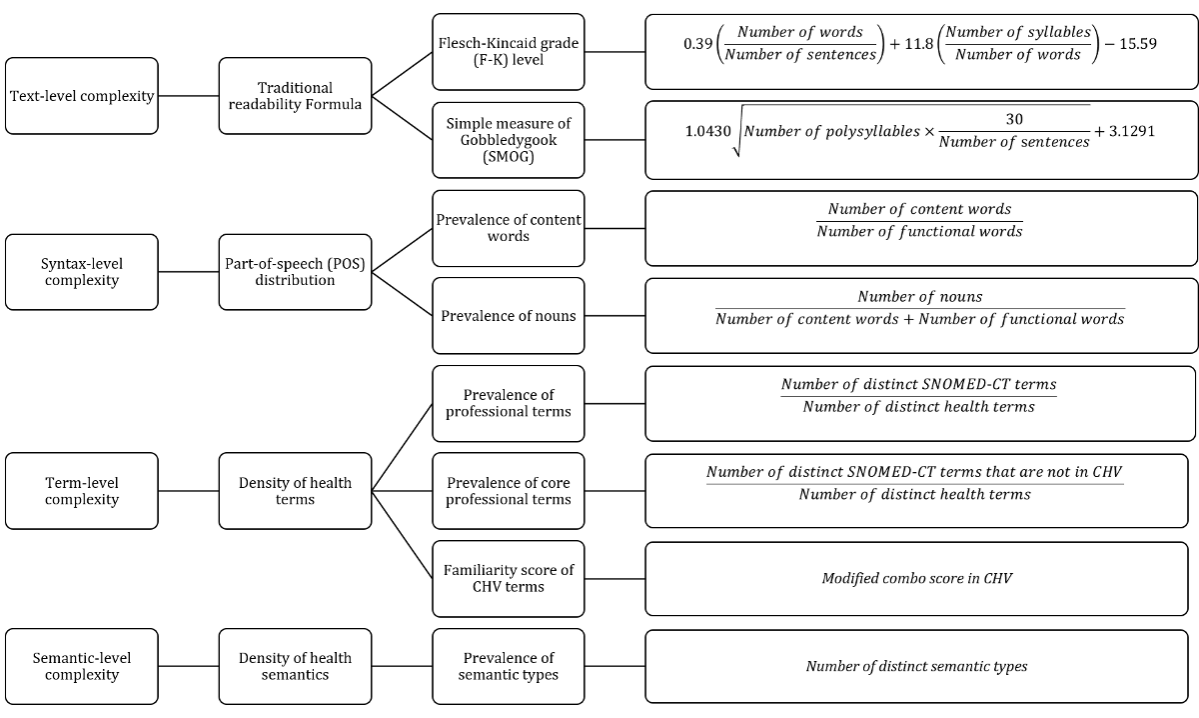
Consumer health language complexity measurement framework (CHELC).

Considering the overlap between lay and professional health terms, we proposed to use the ratio of core professional term coverage, which is the percentage of health terms that are in the Systematized Nomenclature of Medicine-Clinical Terms (SNOMED-CT) but not in consumer health vocabulary (CHV). In total, we included 8 metrics for text-level, syntax-level, term-level, and semantic-level complexity measurements in the proposed framework CHELC (Figure 1).

**Table 1:** Existing metrics for assessing health text complexity.

Health readability measure		Measure specification	Inclusion	Inclusion or exclusion rationale
**Text level**				
	Word length or syllable length [16,28]	Average number of characters (eg, syllables) in a given lexical item	No	Already measured in traditional readability metrics
	Sentence length [16,28]	Average number of words in a sentence	No	Already measured in traditional readability metrics
	Paragraph length [16,28]	Average number of sentences in a paragraph	No	Not applicable for CHL^a^ complexity measure
	Traditional readability metrics [10,25,26,40,41]	Flesch-Kincaid grade level, Simple Measure of Gobbledygook, and Gunning fog	Yes	(1) Well-established formulas that are widely utilized in the literature; (2) Combining word, syllable and sentence length; and (3) Flesch-Kincaid grade and Simple Measure of Gobbledygook are the most used readability metrics
**Syntax level**				
	Ratio of content word [15,42]	Ratio of content words (ie, noun, adjective, verb, and adverb) to functional words (ie, pronoun, determiner, preposition, qualifier, conjunction, interjection)	Yes	Indicator for syntax-level complexity measure; validated in previous literature
	Ratio of nouns [16,42]	Ratio of nouns to all types of parts of speech	Yes	Indicator for syntax-level complexity measure; validated in previous literature
**Term level**				
	Average familiarity score of CHV^b^ [17,28]	Frequency use of each CHV term to the lay people	Yes	Indicator to tell how lay health terms are used in CHL
	Coverage in CHV [15]	Ratio of CHV terms of all terms	No	We used the ratio of professional health terms
	Coverage in basic medical dictionary [16]	Health terms that are in basic medical dictionaries	No	Not applicable for CHL complexity measure
	Coverage in the Unified Medical Language System [15,16]	Ratio of Unified Medical Language System terms	Yes	We utilized the Systematized Nomenclature of Medicine-Clinical Terms as the source of professional health terms
	Term overlap ratio [17]	A higher overlap indicates a more cohesive and easier to read text; overlapped terms/all terms in the document	No	Not applicable for CHL complexity measure
	Vocabulary size [16]	Distinct word counts in the corpus	No	Not applicable for CHL complexity measure
**Style level**				
**Semantic level**				
	Diversity of health topics [15]	Ratio of semantic types indicated in the Unified Medical Language System	Yes	Indicator for semantic-level complexity measure; validated in previous literature

^a^CHL: consumer health language.

^b^CHV: consumer health vocabulary.

#### Text-Level Complexity

Text-level complexity utilizes the length of lexical units (eg, words, sentences, paragraphs) to indicate the lexical complexity of health texts. The unit may change depending on whether the length is applied to words (average number of syllables/characters per word) [16], sentences (average words per sentence) [28], or paragraphs (average sentences per paragraph) [16]. As a commonly used metric, it assumes that longer lexical units require more cognitive loads, thereby making the text more complex. Most studies have utilized one or more readability formulas (eg, the Flesch-Kincaid grade level [F-K] and Simple Measure of Gobbledygook [SMOG]) to assess text-level complexity, in which word length or sentence length are considered in the grade level ranking or level of difficulty of the health texts [10].

For text-level complexity, we applied F-K [43] and SMOG [44] to quantify the text-level complexity of CHL. The F-K formula assigned a grade level to indicate the minimum schooling (grade) readers should have to understand the text. The formula assumes that the higher the average number of syllables and words per sentence there are, the more complex the text is [43]. A grade lower than 5.0 indicates that the text is very easy to comprehend. A grade higher than 12.0 indicates greater difficulty and reading level that requires a college degree or above. Similarly, the SMOG formula considers the number of polysyllabic words [44]. Essentially, the more polysyllabic words, the higher the SMOG score, and the more difficult the texts are.

#### Syntax-Level Complexity

Syntax-level complexity utilizes POS distribution to evaluate the complexity of health texts [29]. In general, there are 10 commonly used POS types in English, which can be categorized into content words (ie, noun, adjective, verb, adverb) and functional words (ie, pronoun, determiner, preposition, qualifier, conjunction, interjection). Every word in the health text can be assigned a POS tag. A higher proportion of noun words or content words indicates more complex health texts [16]. Accordingly, we calculated the ratio of (1) noun words to all POS words and (2) content words to functional words used by each user. We assume that the higher the ratio is, the more complex the CHL is.

#### Term-Level Complexity

Term-level complexity focuses on the complexity related to the density of professional or lay terms (eg, *myocardial infarction* vs *heart attack*). According to health readability research, the more professional terms and fewer lay terms there are, the more complex are the health texts [16]. By mapping terms to existing controlled vocabularies, previous studies have typically measured the term-level complexity with the prevalence of professional terms or lay terms [6,15,16]. Other studies have also utilized the familiarity scores of consumer health terms (provided in CHV) and term cohesiveness (ie, distinct word count or overlapped term ratio) to measure the term-level complexity [16,28,].

To assess the term-level complexity of the health text, we first used the text processing and entity recognition tool MetaMap [45] to extract health terms that belong to 84 out of 127 semantic types in the Unified Medical Language System (UMLS, a compendium of over 190 medical controlled vocabularies) that are relevant to biomedicine, health, and nutrition [46,47]. Then we evaluated the density of professional terms and lay terms by mapping our extracted health terms to 2 controlled vocabularies in the UMLS: CHV and SNOMED-CT. CHV contains a collection of lay health concepts and expressions commonly used by health consumers in their everyday communications [3]. We used the 2015AA version, which includes the latest version of CHV with over 116,324 terms [3]. SNOMED-CT is the world’s largest standardized vocabulary of clinical and medical terms mostly used in health information systems such as electronic health records [48-50]. In this study, CHV was used to evaluate the usage of lay health terms, whereas SNOMED-CT terms were referred to as professional terms. We developed the following 3 measures to evaluate term-level complexity:

- Prevalence of professional terms: we used the ratio of professional terms (number of distinct SNOMED-CT terms) to all health-related terms (number of distinct health terms) to measure the density of professional terms used by each user in a health corpus. We assumed that the higher the ratio is, the more complex is the CHL.
- Prevalence of core professional terms: we first excluded CHV terms from SNOMED-CT terms to obtain the core professional terms (professional health terms that are not commonly used by laypersons), and used the ratio of core professional terms to all health-related terms to measure the density of core professional terms used by each user in a health corpus. We assumed that the higher the ratio is, the more complex is the CHL.
- Familiarity score of CHV terms: it refers to the familiarity of each CHV term to laypersons [17]. It is also referred to as the combo score in CHV, which combines frequency score (term difficulty based on its frequency in several large text corpora), context score (term difficulty based on its context), and Concept Unique Identifier score (term difficulty derived from how it is close to well-known easy and difficult concepts in the UMLS). We used a modified combo score that ignores easy words from the Dale-Chall list [17,51]. The higher the score is, the easier the term is. We calculated the average familiarity score of terms written by each user. We assumed that users using more complex CHL have a relatively low average familiarity score for the CHV terms.

#### Semantic-Level Complexity

Semantic-level complexity refers to the complexity of the diversity of the semantics of health texts. Previous studies have found that if the health text includes more diverse health topics, it is more complex [10]. Operationally, the coverage of semantic types in the UMLS was accounted for semantic-level complexity [47].

We extracted the health terms using MetaMap and counted the average distinct semantic types of the terms used in CHL. We assume that if a user mentioned more distinct semantic types, his or her CHL is more complex.

### Consumer Health Language Complexity Scores

We regarded CHL complexity as a 4-faceted variable, which includes metrics related to text-level, syntax-level, term-level, and semantic-level complexity. Each corpus was represented by a vector of 8 metrics for complexity computation. The values of all 8 metrics were generated for every user in the health corpus.

For each metric, the values for users in all health corpora were ranked [52,53] using the same mechanism of Wu et al [16]. In other words, the ranking value for each metric for users was indicated as the complexity differences among users [54,55]. Except for the familiarity score of CHV terms, the higher the metric value is, the more complex the user’s health language is. It should be noted that we ranked the familiarity score of CHV terms in reverse order. All the missing values of metrics were replaced by the mean of the corresponding metric.

In this proof-of-concept study, each metric in a facet was regarded to contribute equally to the complexity score of that facet. As there is no agreed-upon definition of health text complexity, each facet has equal weight when calculating the overall complexity score (CHELCS_overall_). The idea of aggregating the metrics is that described by Wu et al [16]. We aggregated the ranks of metrics for each facet using standard aggregate functions with the same weights [56]. Other researchers can use different weights for each metric or facet based on their definitions of CHL complexity.

Let *f_ij_* be the *j^th^* observed metric value of the *i^th^* facet and *f’_ij_* be the *j^th^* observed metric value of the specific user whose complexity is calculated in the *i^th^* facet.

The formula of CHELCS_overall_ for every user in the health corpora was as follows:



We defined *r_ij_*, the rank of the *j^th^* metric of the *i^th^* facet, as the number of users whose *f_ij_* is not greater (not smaller for metric *familiarity score of CHV terms*) than *f’_ij_*_._ Note that *m* represents the number of facets, *n_i_* represents the number of metrics in the *i^th^* facet, and *N* is the total number of users.

We calculated the aggregated rank of the metrics for all facets of CHL complexity. We defined *r_ij_*/*N* as the normalized rank ranging from 0 to 1. Then the aggregated complexity score of the *i^th^* facet is calculated as 
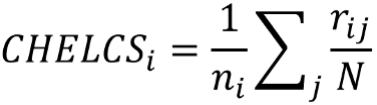
. The overall complexity score of all facets is calculated as 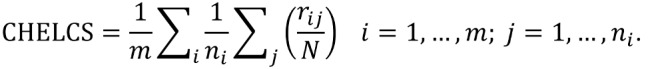 , which is used to represent the overall CHL complexity of every user. All CHELCS range from 0 to 1, and the higher score means the responding user has more complex CHL complexity in all health corpora.

### Data Collection

We utilized CHELC, a complexity measure framework that combines text-level (CHELCS_text_), syntax-level (CHELCS_syntax_), term-level (CHELCS_term_), semantic-level (CHELCS_semantic_), and overall (CHELCS_overall_) complexity scores, to compare the CHLs used in online forums targeting 3 user groups: general public, people with ASD, and deaf and hearing-impaired people. We collected data from various online discussion boards and social media to represent the CHL use of our groups of interest. All 3 data sources in this study were chosen because of their popularity in our interest groups and the convenience of data collection.

We chose AllDeaf [57], a leading online community for deaf and hearing-impaired people who can communicate in English. As of June 2017, AllDeaf had 63,566 members and 114,801 threads. This community has 22 forums in which people can communicate different aspects of everyday life concerns related to deafness, such as sign language, assistive technologies, and health. The majority of the health-related issues are discussed in the forum *Lifestyle, Health, Fitness & Food*. After manually removing the threads that were unrelated to health (eg, food recipes), we retained 1639 threads and 31,006 posts from that forum, which includes health discussions from 2005 to 2016.

Another data source was Wrong Planet [58], which is the main English-language online community developed for people with ASD to discuss everyday life topics. It has 37,350 members and 290,067 threads. Similar to AllDeaf, Wrong Planet has 29 forums. Their users mainly discuss health-related topics in the forum *Health, Fitness & Sports*. After manually removing unrelated threads in that forum, we obtained 2816 threads and 31,194 posts, covering health discussions from 2004 to 2017.

To represent the use of health language by general health consumers, we selected general health discussions in Yahoo! Answers, which is one of the most popular social Q&A sites used by people to discuss health and other life topics. To make the sample size comparable to those collected from AllDeaf and Wrong Planet, we generated a random sample of 8000 questions and their respective answers in the health category, resulting in 34,048 posts from 2009 to 2014.

### Data Processing and Analysis

We extracted health-related posts in the 3 forums and calculated CHELCS for each user using text-level (CHELCS_text_), syntax-level (CHELCS_syntax_), term-level (CHELCS_term_), semantic-level (CHELCS_semantic_), and overall (CHELCS_overall_) complexity. As it is not feasible to analyze behavioral patterns for users contributing to few discussions, we only analyzed posts from users who contributed more than 4 sentences per post on average. For the term-level analysis, we only included users who used more than 20 distinct health terms per post. For text- and syntax-level metrics, we generated the scores for each post through a Web-based readability measurement tool [59] and then calculated the complexity score for each user in the 3 corpora using a rank-based algorithm. For the term coverage and semantic analysis, we analyzed the data in MySQL (Oracle Corporation) and Microsoft Excel. We visualized the distributions using CHELCS_text_, CHELCS_syntax_, CHELCS_term_, CHELCS_semantic_, and CHELCS_overall_ for users in each group in Microsoft Excel. Then we employed a 2-sample Kolmogorov-Smirnov test (K-S test) to determine if the CHELCS of the various groups were significantly different. We conducted an analysis of covariance (ANCOVA) to control for possible impacts of sentence number per post on CHELCS when comparing CHL complexity scores of the 3 groups. More detailed comparison results of 3 groups in 8 metrics were presented in Multimedia Appendix 1, and correlations of CHELCS scores were analyzed in Multimedia Appendix 2. K-S test and ANCOVA were performed in R software (The R Foundation for Statistical Computing).

## Results

### Basic Characteristics of the Corpora

As seen in Table 2, although we extracted similar numbers of posts from the 3 corpora regardless of the number of sentences, the numbers of posts with more than 4 sentences were different among the 3 groups. Compared with the other online forums, Yahoo! Answers had the fewest number of posts, the most threads, and involved the most users, but had the least number of distinct health terms contributed by the average user. This might be because of the differences between specialized online forums that are closed communities and general social Q&A sites that are open to the public [60]. However, the 3 corpora did not have major differences in the number of sentences, sentence lengths, and word lengths, implying that platform differences would not significantly impact the overall CHL used in each community. The 3 user groups shared 68 out of 84 health semantic types in the UMLS.

**Table 2:** Basic textual characteristics of the 3 health corpora.

Basic textual characters	Health corpora		
	AllDeaf (deaf and hearing-impaired people), n	Wrong Planet (people with ASD), n	Yahoo! Answers (general public), n
Number of posts	27,545	26,484	12,560
Number of threads	1623	2751	3756
Number of involved users	788	2978	9544
Average number of sentences per post per user	9.21	9.15	9.63
Average number of words per sentence per user	12.14	13.99	13.09
Average number of syllables per word per user	1.37	1.41	1.35
Average number of letters per word per user	4.14	4.23	4.11
Distinct health terms per user	199.87	91.63	39.09
Mentioned semantics number	71	71	72

### Text-Level Complexity

**Figure 2:**
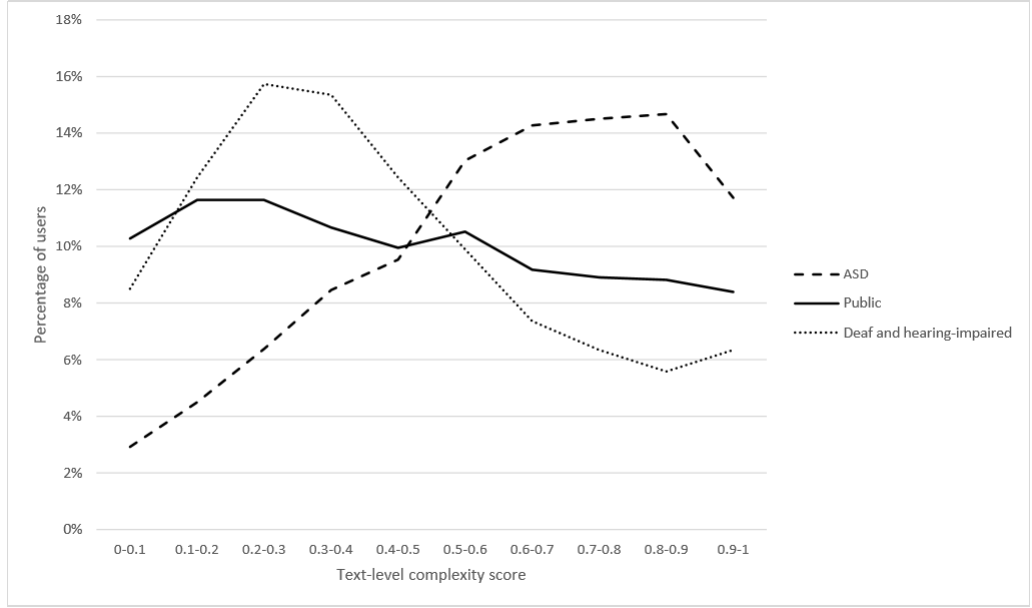
Text-level complexity comparison for users in the 3 health corpora. ASD: autism spectrum disorder.

The CHELCS_text_, which ranges from 0 to 1, indicates the text-level complexity ranking of the individual user among all users in the 3 online forums. Figure 2 shows the distribution of text-level complexity scores of users in 3 corpora.

The 2-sample K-S test results indicate CHELCS_text_ scores of people with ASD, deaf and hearing-impaired people, and the general public were significantly different (D_d-a_=0.332, *P*_d-a_<.001; D_d-p_=0.108, *P*_d-p_<.001; D_a-p_=0.228, *P*_a-p_<.001 [d-a refers to score comparison between *CHELCS_text_ of deaf and hearing-impaired users* and *CHELCS_text_ of users with ASD*; d-p refers to score comparison between *CHELCS_text_ of the deaf and hearing-impaired users* and *CHELCS_text_ of the general public*; a-p refers to score comparison between *CHELCS_text_ of users* *with ASD* and *CHELCS_text_ of the general public*] ). As seen in Figure 2, most deaf and hearing-impaired users wrote texts with lower complexity, whereas users with ASD used more complex texts in their posts. General public users did not significantly differ in their use of polysyllabic words.

After controlling for the number of sentences per post, the ANCOVA results (*F*_2_=304.5; *P*<.001) show that users with ASD (mean 0.606) used significantly more complex texts than the other 2 groups (*P*<.001) and the general public used significantly more complex texts (mean 0.473) than those in the deaf and hearing-impaired group (mean 0.431; *P*<.001).

### Syntax-Level Complexity

**Figure 3:**
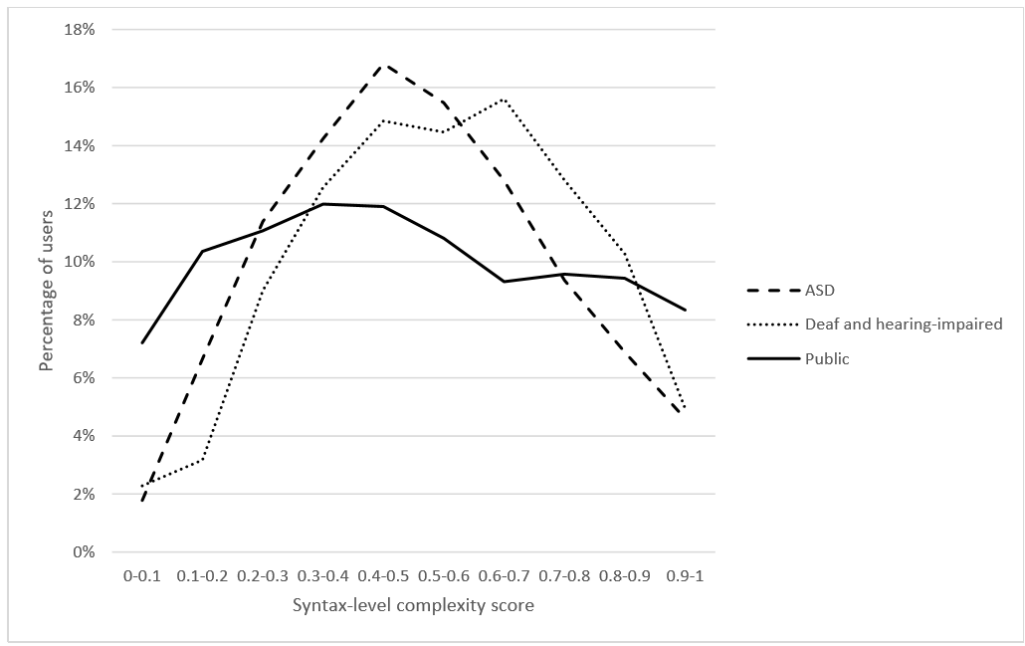
Syntax-level complexity comparison for users in the 3 health corpora. ASD: autism spectrum disorder.

The CHELCS_syntax_ indicates complexity ranking related to the prevalence of content words, especially nouns. As seen in Figure 3, the peak CHELCS_syntax_ scores for deaf and hearing-impaired users ranged from 0.6 to 0.7, whereas the peak CHELCS_syntax_ scores for users with ASD ranged from 0.4 to 0.5. Regarding general public users, they did not show a clear syntax complexity preference. The two-sample K-S tests indicate that CHELCS_syntax_ scores were significantly different (D_d-a_=0.108, *P*_d-a_<.001; D_d-p_=0.153, *P*_d-p_<.001; D_a-p_=0.098, *P*_a-p_<.001).

After controlling for the number of sentences per post, the results (*F*_2_=19.206; *P*<.001) show that deaf and hearing-impaired users used (mean 0.551) significantly more complex syntax than those in the other 2 groups (*P*<.001), whereas usage of complex syntax was not significantly different between users with ASD (mean 0.506) and the general public (mean 0.494; *P*=.07).

### Term-Level Complexity

**Figure 4:**
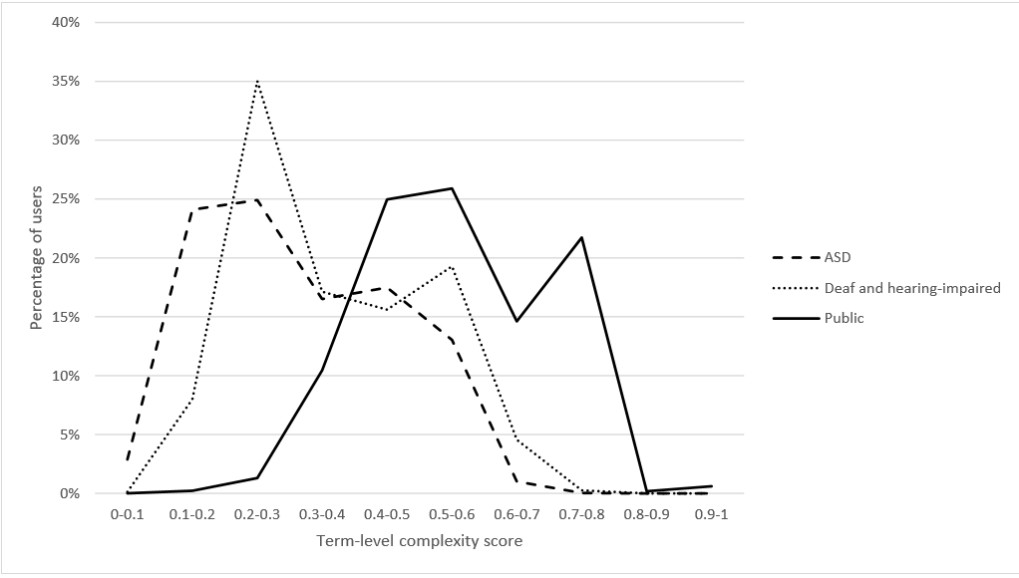
Term-level complexity comparison for users in the 3 health corpora. ASD: autism spectrum disorder.

The CHELCS_term_ focuses on the complexity of the health terms used in each forum. As seen in Figure 4, bimodal distributions were observed in all 3 corpora. Most general public users had relatively higher CHELCS_term_ ranging from 0.2 to 0.9, whereas most users in the other 2 groups had complexity scores lower than 0.7. The two-sample K-S test results indicate that the CHELCS_term_ scores of users with ASD, deaf and hearing-impaired, and general public users were significantly different in the prevalence of professional terms (D_d-a_=0.208, *P*_d-a_=.009; D_d-p_=0.523, *P*_d-p_<.001; D_a-p_=0.590, *P*_a-p_<.001).

After controlling for the number of sentences per post, the ANCOVA results (*F*_2_=3822.320; *P*<.001) show that the general public users (mean 0.568) used significantly more complex health terms than those in the other 2 groups (*P*<.001), and deaf and hearing-impaired users (mean 0.370) used more complex terms than users with ASD (mean 0.316; *P*<.001).

### Semantic-Level Complexity

**Figure 5:**
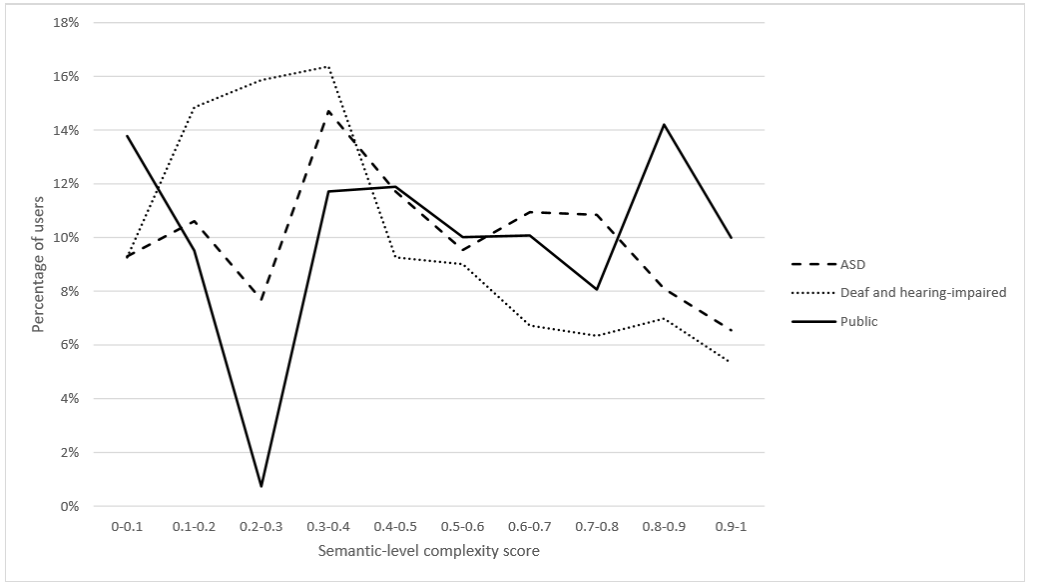
Semantic-level complexity comparison for users in the 3 health corpora. ASD: autism spectrum disorder.

The CHELCS_semantic_ indicates the diversity of semantic types. Figure 5 shows the distribution of the semantic-level complexity scores in the 3 groups. The two-sample K-S test results indicate that the CHELCS_semantic_ scores for the 3 groups were significantly different (D_d-a_=0.141, *P*_d-a_<.001; D_d-p_=0.215, *P*_d-p_<.001; D_a-p_=0.116, *P*_a-p_<.001). As all health corpora were from social media platforms, the semantics that people utilized might be more influenced by the context than personal health literacy.

By controlling the number of sentences per post, results (*F*_2_=53.082; *P*<.001) show that, on average, general public users (mean 0.514) used more semantic types than those in the other 2 groups (*P*<.001). Users with ASD (mean 0.478) included more semantic types than deaf and hearing-impaired users (mean 0.416; *P*<.001). In essence, general public users mentioned more diverse health topics than users with ASD and deaf and hearing-impaired users.

### Overall Complexity

**Figure 6:**
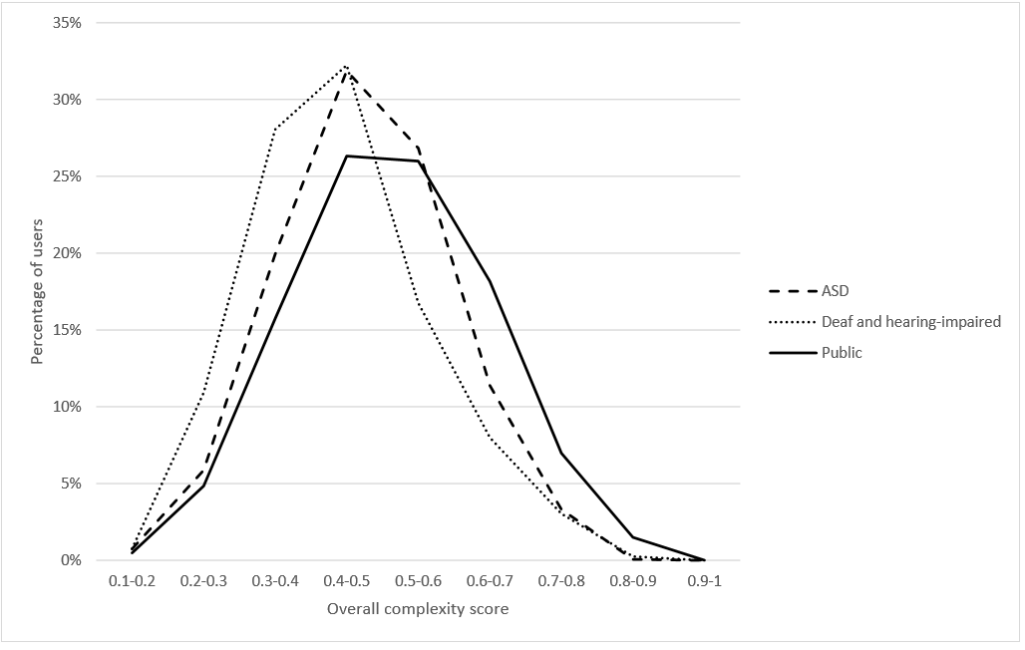
Overall complexity comparison for users in the 3 health corpora. ASD: autism spectrum disorder.

Figure 6 shows the CHELCS_overall_ for users in the 3 forums. The two-sample K-S test results indicate that the overall CHL complexity scores for users in the 3 corpora were significantly different (D_d-a_=0.171, *P*_d-a_<.001; D_d-p_=0.250, *P*_d-p_<.001; D_a-p_=0.129, *P*_a-p_<.001).

After controlling the number of sentences for each participant, the ANCOVA result (*F*_2_=167.748; *P*<.001) shows that, on average, general public users (mean 0.512) had more complex CHL than the other 2 groups (*P*<.001). Users with ASD (mean 0.476) had more complex CHL than deaf and hearing-impaired users (mean 0.442; *P*<.001).

## Discussion

### Principal Findings

As health information on the Web often contains medical jargon and complex sentences, general health consumers often find it hard to search for and understand Web-based health information [17]. We argue that health text complexity measurements need to measure the complexity of various CHLs to inform content providers to tailor health information on the Web for health consumers with varying CHL preferences [20,36]. To this end, we developed CHELCS to quantify CHL complexity differences. We applied this measurement to examine CHL complexity differences of health-related posts in 3 online forums targeting the general public, people with ASD, and deaf and hearing-impaired people. In particular, we collected user-generated discussions from 3 online health communities: Yahoo! Answers, Wrong Planet, and AllDeaf. We calculated 8 health readability metrics for each post in the 3 online forums, and calculated text-level (CHELCS_text_), syntax-level (CHELCS_syntax_), term-level (CHELCS_term_), semantic-level (CHELCS_semantic_), and overall (CHELCS_overall_) complexity scores. We then compared the CHL complexity differences for the 3 user groups based on these 5 complexity scores (CHELCS).

The results supported that CHLs of the 3 user groups were significantly different. General public users used more complex health terms and more diverse semantics compared with users with ASD and deaf and hearing-impaired users. Consistent with previous findings, users with ASD used words with more syllables, fewer content or noun words, and less complex health terms [35,38]. Deaf and hearing-impaired users used more content words or nouns, fewer complex words, and less diverse semantics [34,36]. CHELCS results indicated that overall, general public users used more complex CHL than those in the other 2 groups. Overall, the findings from CHELCS measurement were consistent with previous findings of CHL differences among people with ASD, deaf and hearing-impaired people, and public groups.

On the basis of our results, when developing algorithms to simplify health content for different user groups, we need to use more lay health terms for deaf and hearing-impaired users and for users with ASD, less complex words for deaf and hearing-impaired users, and more functional words for users with ASD. For example, as the average F-K grade of MedlinePlus articles is around 8 to 10 [15,16], deaf and hearing-impaired users may need more textual simplifications than the other 2 groups.

To the best of our knowledge, this is the first framework that harnesses consumer-generated textual data to assess the complexity of language that they are comfortable using in their health communications. An understanding of the various CHL complexities of different user groups can provide better insights for the development of adaptive readability assessment tools and adaptive text simplification services.

### Limitations

Some limitations should be noted. We could not filter out all the users who are not deaf and hearing impaired or users with ASD, which might affect our findings of the 3 user groups to a certain extent. The data were collected from 3 nontopic–specific health forums. The impact of health topics on text complexity was not controlled in this exploratory study. For example, CHLs by patients with chronic conditions may be more complex than the average healthy consumers. As the average user contributed little text content in the forums, the findings might not fully depict the language complexity preference of each user. More datasets, such as patient blogs and social media, need to be explored in future studies.

In this proof-of-concept study, the framework CHELC was developed with 8 metrics validated in previous health readability studies to compare CHL complexity differences. Although these metrics have been validated in previous studies, to the best of our knowledge, they have not been used to compare CHLs of different consumer groups. With a lack of research in this field, there is no agreed-upon definition of CHL complexity with respect to different aspects. Therefore, we cannot find a ground truth dataset or standard to validate CHELCS when estimating CHL complexity differences. In this exploratory study, the evaluation of CHELCS was based on previous research findings of the 3 groups in terms of their language complexity preferences. Although our results were consistent with previous findings, this framework and complexity scores are more informative than conclusive. For example, the scores will be different if more metrics are included in this framework, or if the weights of different metrics are defined differently. Also, to more accurately estimate adaptive simplification efforts, it is critical that future studies further assess the CHELCS difference between Web-based consumer health information sources and various CHLs.

### Conclusions

The results of this study demonstrate that differences exist among health consumers with respect to the complexity of their language use when discussing health-related topics. A complexity measurement framework (CHELC) and its accompanying scores (CHELCS) were developed to quantify CHL complexity differences among different user groups. Future studies could further apply CHELCS to other datasets from different user groups. Specifically, there is a clear need for the research on understanding CHL complexity differences that translates to adaptive simplification services for different user groups.

## Appendix

Multimedia Appendix 1 Complexity score of seven metrics in consumer health language complexity scores.

Multimedia Appendix 2 Correlations of consumer health language complexity scores in health corpora.

## Abbreviations

ANCOVA: analysis of covariance
ASD: autism spectrum disorder
CHELC: consumer health language complexity
CHELCS: consumer health language complexity scores
CHL: consumer health language
CHV: consumer health vocabulary
F-K: Flesch-Kincaid grade level
K-S: Kolmogorov-Smirnov
NIH: National Institutes of Health
POS: parts of speech
Q&A: question and answer
SNOMED-CT: Systematized Nomenclature of Medicine-Clinical Terms
UMLS: Unified Medical Language System
