# Supplementary material for: An Informatics Framework to Assess Consumer Health Language Complexity Differences: Proof-of-Concept Study
Source: J Med Internet Res. 2020 May 21;22(5):e16795. doi: 10.2196/16795 (PMC7273233; doi:10.2196/16795)
Supplement: Multimedia Appendix 1 [file jmir_v22i5e16795_app1.pdf]

Multimedia Appendix 1: Complexity Scores of Seven Metrics in CHELCS

In the paper, we proposed a health text complexity measurement framework CHELC to compare consumer health languages (CHLs). We incorporated eight validated metrics from previous literature, each of which measures text-level, syntax-level, term-level, or semantic-level complexity of health texts. In the main texts, we utilized our proposed tool to compare $CHELCS_{overall}$, $CHELCS_{text}$, $CHELCS_{syntax}$, $CHELCS_{term}$, and $CHELCS_{semantic}$ of the public, deaf and hearing-impaired users, and ASD users on social media. Except for $CHELCS_{semantic}$, other complexity scores were composed of more than one metric. To deeply understand how CHLs are different for three groups, this appendix shows the detailed comparison results for seven metrics respectively (except for density of semantic types).

Text-level complexity

$CHELCS_{text}$ was composed of Flesch-Kincaid Grade Level (F-K score) and Simple Measure of Gobbledygook (SMOG). As seen in Figure 1, peak ASD users utilized complex texts with F-K score ranging from 0.7 to 0.9, whereas peak deaf and hearing-impaired users preferred simple texts with F-K score from 0.2 to 0.4. In comparison, the uniform distribution of the public indicated that general public users do not show an overall preference over complex or easy texts. The two-sample K-S test results indicated that the distributions of ASD, deaf and hearing-impaired, and general public individuals were significantly different in F-K score ($D_{d\text{-}a}$=0.313, $P_{d\text{-}a}$<.001; $D_{d\text{-}p}$=0.103, $P_{d\text{-}p}$<.001; $D_{a\text{-}p}$=0.225, $P_{a\text{-}p}$<.001; [d-a refers to scores comparison between deaf and hearing impaired and ASD users; d-p refers to scores comparison between deaf and hearing impaired users and the general public; a-p refers to scores comparison between ASD users and the general public]).

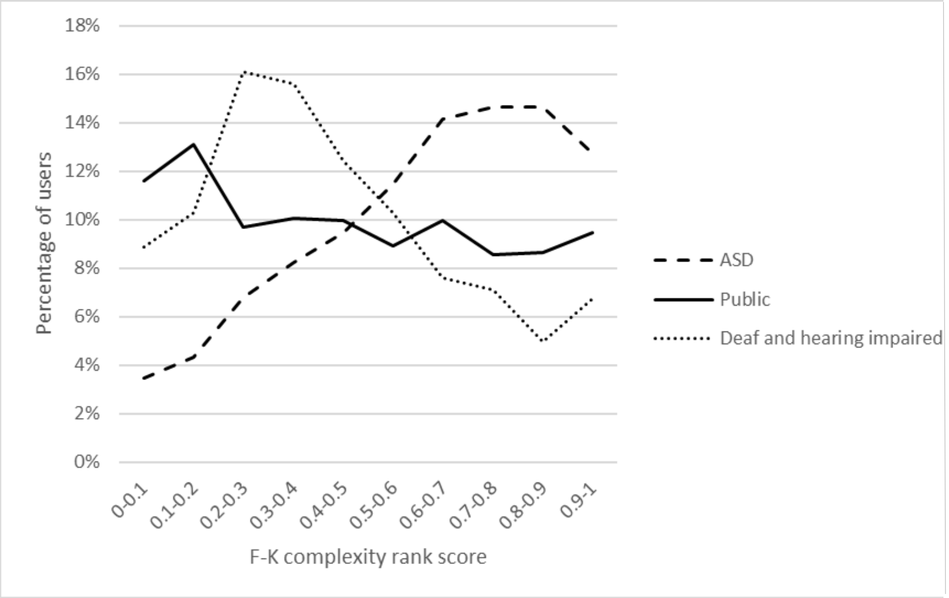

Figure 1. F-K Score comparison for users in the 3 health corpora. ASD: autism spectrum disorder

By controlling the number of sentences per post, the ANCOVA results (F=287.2; df=2; P<0.001) show that ASD users (mean=0.608) used significantly more complex words than other two groups (P<.001); and general public (mean=0.471) used significantly more complex words than deaf and hearing-impaired group (mean=0.440; P=0.01).

Comparison results of SMOG are illustrated in Figure 2. Bimodal distributions were observed in deaf and hearing-impaired and ASD groups, whereas multimodal distribution was found for the public. Similar to F-K score results, general public users do not show an overall pattern of text complexity preference; whereas ASD and deaf and hearing-impaired users show the opposite patterns of complexity preference. The two-sample K-S test results indicate that the distributions of ASD, deaf and hearing-impaired, and general public users were significantly different in SMOG ($D_{d\text{-}a}$=0.327, $P_{d\text{-}a}$<.001; $D_{d\text{-}p}$=0.134, $P_{d\text{-}p}$<.001; $D_{a\text{-}p}$=0.231, $P_{a\text{-}p}$<.001).

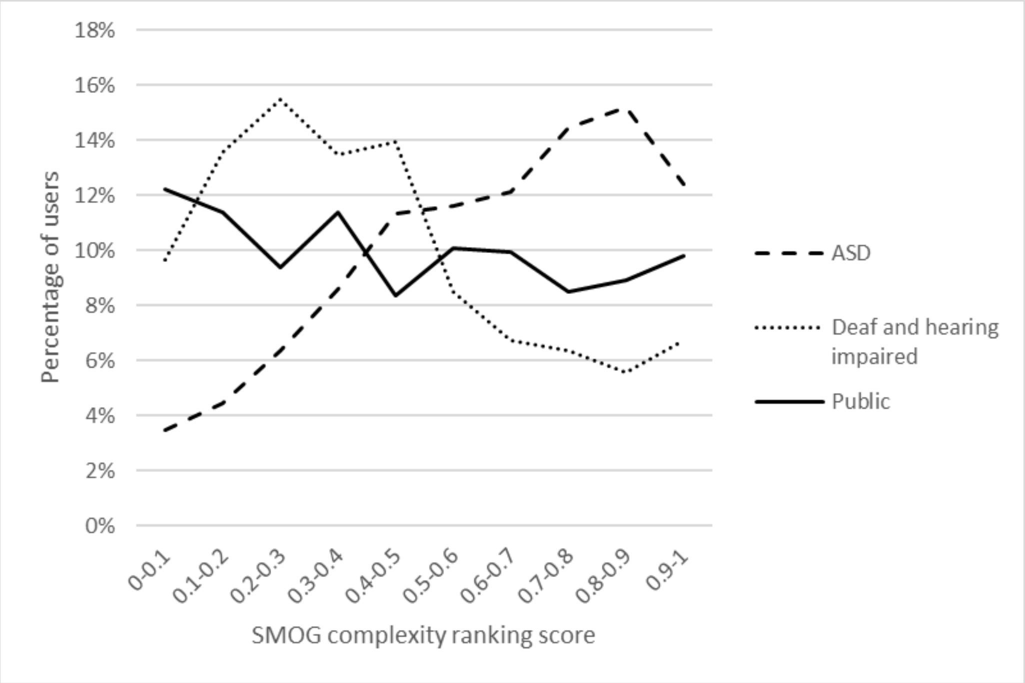

Figure 2. SMOG complexity comparison for users in the 3 health corpora. ASD: autism spectrum disorder

By controlling the number of sentences per post, the ANCOVA results (F=286.1; df=2; P<.001) show that ASD users (mean=0.605) used significantly more complex words than other two groups (P<.001); and the general public (mean=0.474) used significantly more complex words than deaf and hearing-impaired users (mean=0.422; P<.001).

Syntax-level complexity

Prevalence of content words and nouns were metrics measuring $CHELCS_{syntax}$. As seen in Figure 3, bell curves were observed for deaf and hearing-impaired and ASD users, whereas well curve was found for the public: public health consumers on social media flock to use high or low prevalence of content words, whereas the other two groups tend to use content words with mid-

sized prevalence. The two-sample K-S test results indicate that the distributions of ASD, deaf and hearing-impaired, and general public individuals were significantly different in the prevalence of content words ($D_{d\text{-}a}$=0.07, $P_{d\text{-}a}$=0.006; $D_{d\text{-}p}$=0.145, $P_{d\text{-}p}$<.001; $D_{a\text{-}p}$=0.112, $P_{a\text{-}p}$<.001).

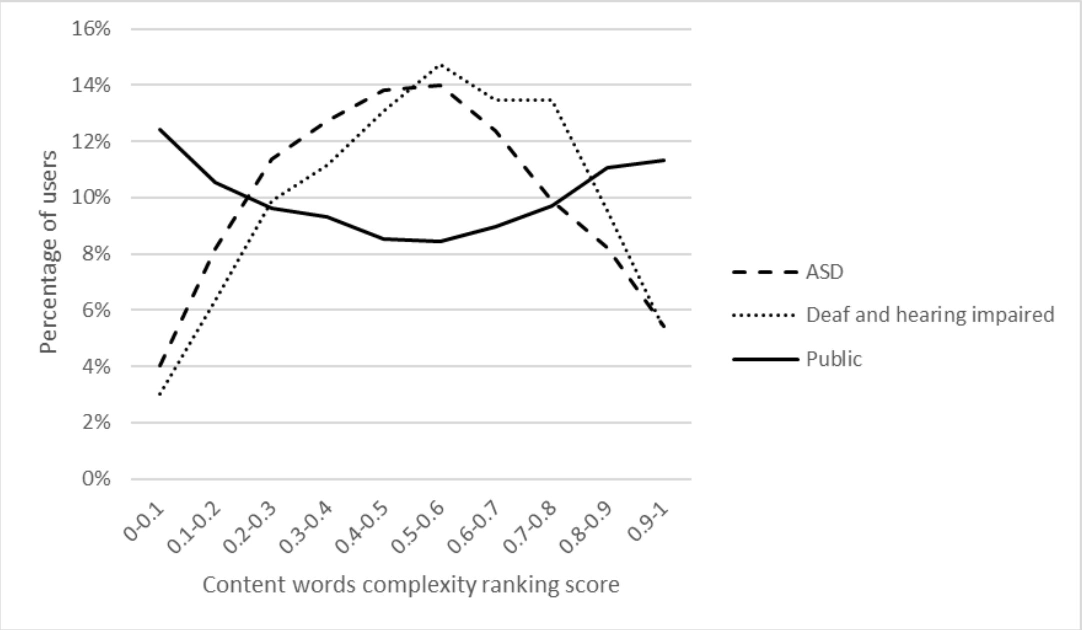

Figure 3. Content words complexity comparison for users in the 3 health corpora. ASD: autism spectrum disorder

By controlling the number of sentences per post, the ANCOVA results (*F*=6.0; *df*=2; *P*=0.003) show that deaf and hearing-impaired users (mean=0.532) used significantly more content words than other two groups (P_{ASD}=0.03; P_{public}=0.002); but the difference between the general public (mean=0.497) and ASD group was not significant (mean=0.503; *P*=0.851).

Noun prevalence comparison results were found in Figure 4. Bimodal distribution was observed for deaf and hearing-impaired, bell curve for ASD, and well curve for the public. Patterns for three groups to use noun words were similar to those of content words, except for ASD users having two peaks not in the middle. The two-sample K-S test results indicate that the distributions of ASD, deaf and hearing-impaired, and general public users were significantly different in the prevalence of nouns ($D_{d\text{-}a}$=0.138, $P_{d\text{-}a}$<.001; $D_{d\text{-}p}$=0.190, $P_{d\text{-}p}$<.001; $D_{a\text{-}p}$=0.112, $P_{a\text{-}p}$<.001).

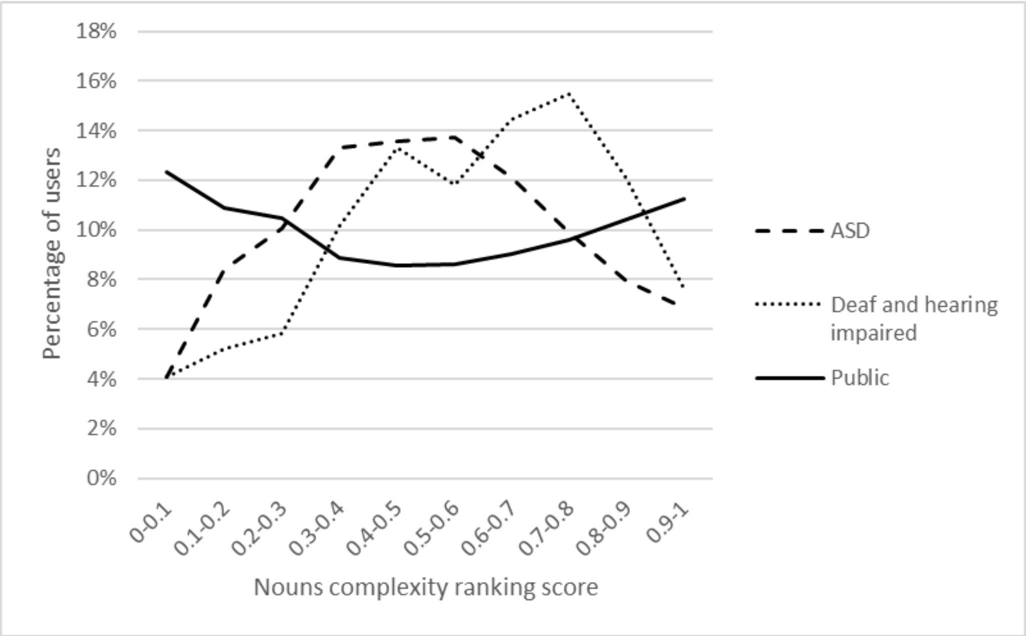

Figure 4. Nouns complexity comparison for users in the 3 health corpora. ASD: autism spectrum disorder

By controlling the number of sentences per post, the ANCOVA results (F=286.1; df=2; P<.001) show that deaf and hearing-impaired users (mean=0.570) used significantly more nouns than other two groups (P<.001); and ASD (mean=0.509) used significantly more nouns than the general public (mean=0.492; P<.001).

Term-level complexity

$CHELCS_{term}$ was composed of prevalence of professional health term, professional core health terms and familiarity score. focused on complexity of the health terms used in health corpora. As seen in Figure 5, multimodal distributions were observed in all three corpora: for deaf and hearing-impaired and ASD users, three peaks were found in 0.3-0.4, 0.5-0.6, and 0.8-0.9; in comparison, most general public users utilized mostly professional health terms ranging from 0.8-0.9. The two-sample K-S test results indicate that the distributions of ASD, deaf and hearing-impaired, and general public users were significantly different in the prevalence of professional health terms ($D_{d\text{-}a}$=0.067, $P_{d\text{-}a}$=0.007; $D_{d\text{-}p}$=0.244, $P_{d\text{-}p}$<.001; $D_{a\text{-}p}$=0.186, $P_{a\text{-}p}$<.001).

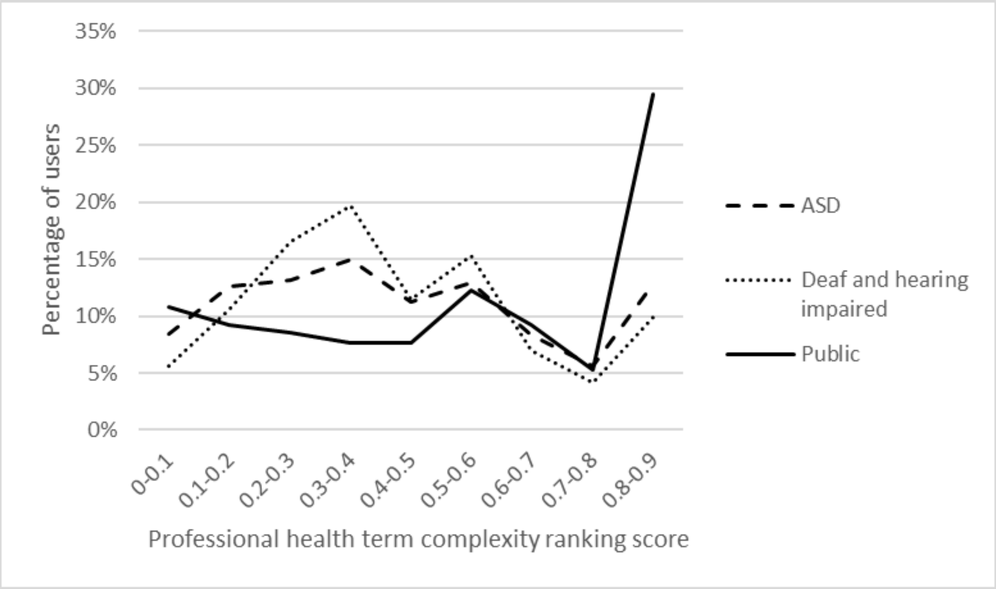

Figure 5. Professional health term complexity comparison for users in the 3 health corpora. ASD: autism spectrum disorder

By controlling the number of sentences per post, the ANCOVA results (F=130.5; df=2; P<.001) show that general public (mean=0.525) used significantly more complex words than other two groups (P<.001); and the difference between ASD (mean=0.440) and deaf and hearing-impaired (mean=0.431) was not significant (P=1.0).

As seen in Figure 6, distributions in all three corpora were different: bell curve was found for deaf and hearing-impaired users with the peak value ranging from 0.3 to 0.4, and bimodal distribution was observed for the general public with two peak value ranges of high complexity ranking score. In comparison, ASD users flock to utilize least core professional health terms less than 0.1. The two-sample K-S test results indicate that the distributions of ASD, deaf and hearing-impaired users, and general public were significantly different in the prevalence of core professional terms ($D_{d\text{-}a}$=0.578, $P_{d\text{-}a}$<.001; $D_{d\text{-}p}$=0.560, $P_{d\text{-}p}$<.001; $D_{a\text{-}p}$=0.651, $P_{a\text{-}p}$<.001).

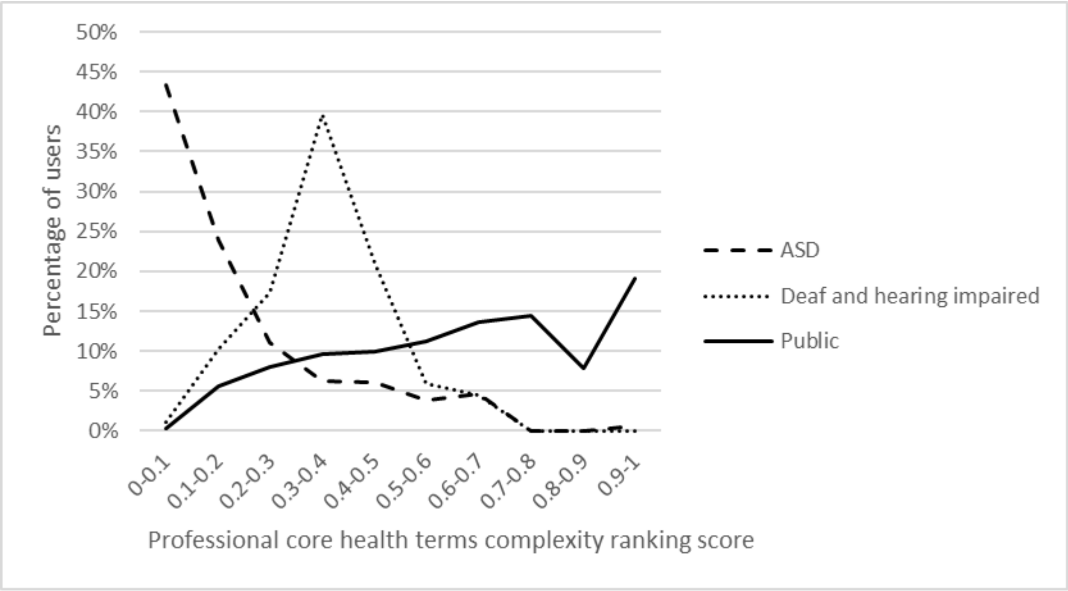

Figure 6. Professional core health term complexity comparison for users in the 3 health corpora. ASD: autism spectrum disorder

By controlling the number of sentences per post, the ANCOVA results (F=4460.2; df=2; P<.001) show that the general public (mean=0.609) used significantly more complex words than other two groups (P<.001); and deaf and hearing-impaired users (mean=0.361) used significantly more professional core health terms than ASD users (mean=0.188, P<.001).

Since most users did not generate more than 20 CHV terms, missing values were replaced with the average value of each health corpus. As seen in Figure 7, more than 90% of the general public users utilized CHV terms with medium complexity. In comparison, peaks of deaf and hearing-impaired and ASD users either chose CHV terms with the low complexity (ranging from 0.1 to 0.2) or highest complexity (ranging from 0.9 to 1). The two-sample K-S test results indicate that the distributions of ASD, deaf and hearing-impaired users, and general public were significantly different in the prevalence of familiarity score (D_{d-a}=0.427, P_{d-a}<.001; D_{d-p}=0.708, P_{d-p}<.001; D_{a-p}=0.746, P_{a-p}<.001).

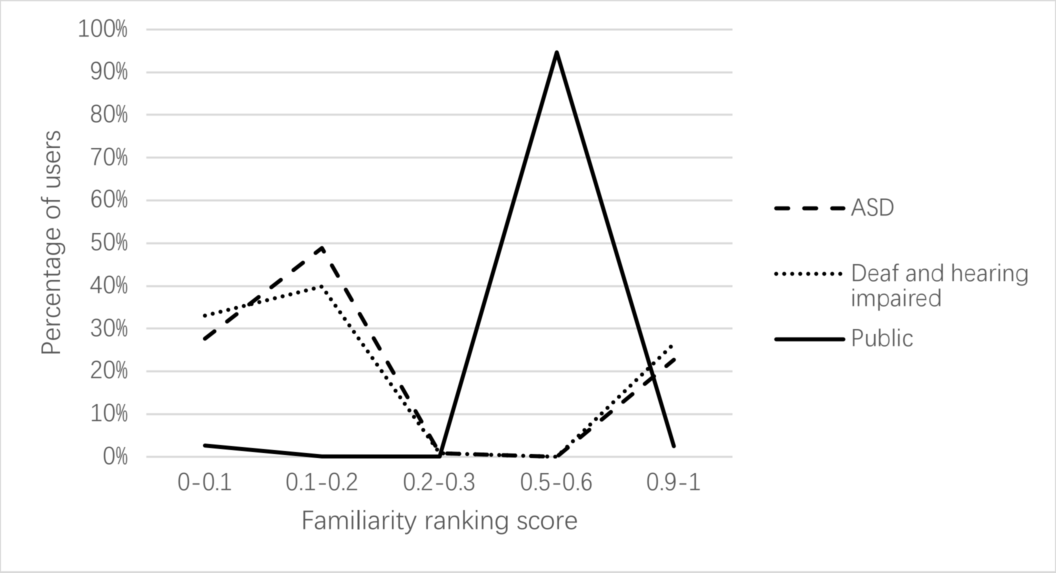

Figure 7. Familiarity score complexity comparison for users in the 3 health corpora. ASD: autism spectrum disorder

By controlling the number of sentences per post, the ANCOVA results (F=1929.6; df=2; P<.001) show that the general public (mean=0.571) used significantly more complex CHV terms than other two groups (P<.001); and the difference between ASD (mean=0.320) and deaf and hearing-impaired (mean=0.317) was not significant (P=1.0).

Summary

After the detailed analyses of seven metrics, we found that metrics within $CHELCS_{text}$ and $CHELCS_{syntax}$ shared similar distributions: for text complexity, the general public do not show a strong preference over easy or complex texts; whereas ASD users preferred more complex and deaf and hearing-impaired users preferred easier texts. For both syntax complexity metrics, bell curves were observed for ASD and deaf and hearing-impaired users, whereas well curve was found for the public. However, distributions of three $CHELCS_{term}$ metrics were all different for three groups, indicating more complex patterns were found for their health term use. In general, ASD users used longer texts with more syllables, and deaf and hearing-impaired users preferred using more content words, especially nouns in their CHL. For health term complexity, the general public used much more complex health terms, core health terms, and CHV terms than two disabled groups; the difference between deaf and hearing-impaired and ASD users was not significant in terms of familiarity score and professional health terms, whereas deaf and hearing-impaired users used more core professional health terms than ASD users.
