# Supplementary material for: An Informatics Framework to Assess Consumer Health Language Complexity Differences: Proof-of-Concept Study
Source: J Med Internet Res. 2020 May 21;22(5):e16795. doi: 10.2196/16795 (PMC7273233; doi:10.2196/16795)
Supplement: Multimedia Appendix 2 [file jmir_v22i5e16795_app2.pdf]

Multimedia Appendix 2: Correlations of CHELCS in Health Corpora

As seen in Multimedia Appendix 2 Table 1, after controlling for the sentence number per post, the Pearson correlation analyses show that most CHELCS scores are weakly correlated (around or less than 0.1) in three health corpora, indicating that our data supported that $CHELCS_{text}$, $CHELCS_{syntax}$, and $CHELCS_{semantic}$ measure different facets of the health text complexity.

Table 1. Correlation matrix of CHELCS in the 3 health corpora

		$CHELCS_{text}$	$CHELCS_{syntax}$	$CHELCS_{term}$	$CHELCS_{semantic}$	$CHELCS_{overall}$
$CHELCS_{text}$	Deaf and hearing impaired	1	-.160***	.076*	.381***	.680***
	ASD	1	-.089***	-.081***	.402***	.679***
	Public	1	-.019	-.233***	.428***	.691***
$CHELCS_{syntax}$	Deaf and hearing impaired		1	.125***	-.105**	.388***
	ASD		1	.103***	-.067***	.442***
	Public		1	-.033***	.037***	.524***
$CHELCS_{term}$	Deaf and hearing impaired			1	.058	.438***
	ASD			1	-.126***	.265***
	Public			1	-.300***	-.018
$CHELCS_{semantic}$	Deaf and hearing impaired				1	.642***
	ASD				1	.649***
	Public				1	.684***
CHELCS	Deaf and hearing impaired					1
	ASD					1
	Public					1

Note: 1. *, **, and *** stand for p-value significance levels of 0.05, 0.01, and 0.001 respectively; 2. ASD: autism spectrum disorder

Strong correlations were observed between $CHELCS_{semantic}$ and $CHELCS_{text}$ for three corpora, and between $CHELCS_{term}$ and $CHELCS_{semantic}$ for the general public users. Strong correlations between $CHELCS_{semantic}$ and $CHELCS_{text}$ indicate that the people tend to discuss contents with more semantics in complex sentences. Further studies are needed to determine why strong correlation between $CHELCS_{term}$ and $CHELCS_{semantic}$ was only found for the general public.

$CHELCS_{overall}$ is strongly correlated with four faceted scores throughout three corpora, except for $CHELCS_{term}$ of the general public. The detailed correlation results for three groups are

different, especially in $CHELCS_{term}$. Further studies (e.g., collecting data from blogs, other online communities) are needed to clarify whether the difference comes from CHL differences of three user groups, or comes from the impact of the platform of social media.
